# Shifts in amino acids profile in chronic pancreatitis

**DOI:** 10.1371/journal.pone.0341087

**Published:** 2026-01-20

**Authors:** Marko Malvik, Kalle Kilk, Raili Müller, Riina Salupere, Mihkel Zilmer, Margus Lember

**Affiliations:** 1 Department of Internal Medicine, Institute of Clinical Medicine, University of Tartu, Tartu, Estonia; 2 Department of Gastroenterology, Tartu University Hospital, Tartu, Estonia; 3 Department of Biochemistry, Institute of Bio- and Translational Medicine, University of Tartu, Tartu, Estonia; 4 Department of Internal Medicine, Endocrinology and Rheumatology, Tartu University Hospital, Tartu, Estonia; University of Life Sciences in Lublin, POLAND

## Abstract

**Background:**

Chronic pancreatitis (CP) can lead to pancreatic exocrine insufficiency (PEI), causing protein malabsorption and altered amino acid levels. Our aim is to investigate amino acid levels in patients with CP and to assess the associations with exocrine insufficiency, pancreatic elastography, and the severity of CP.

**Methods:**

Twenty-five patients with CP were prospectively enrolled in this study. A control group included 28 age- and gender-matched subjects without pancreatic disease. Amino acids were quantified using the Biocrates MxP Quant 500 kit. Differences in amino acid levels were calculated using the non-parametric Wilcoxon test. Pancreatic stiffness was evaluated by EUS-elastography, PEI was assessed using fecal elastase-1 levels. The severity of CP was determined using the M-ANNHEIM classification.

**Results:**

The ratio of nonessential amino acids to essential amino acids, glutamic acid and aspartic acid levels were significantly increased in the CP group, whereas tryptophan levels were significantly decreased in CP. A positive correlation between proteinogenic amino acids and the severity of pancreatitis was found. Proline levels showed a strong correlation with the CP severity index (r = 0.63), a significant correlation with elastase-1 values (r = −0.48, p = 0.02), and a moderate correlation with pancreatic stiffness (r = 0.47, p = 0.09).

**Conclusion:**

Patients with CP have altered amino acid blood levels, an increased ratio of nonessential to essential amino acids. The severity of CP was found to increase the levels of nonessential amino acids, with proline showing the strongest associations with severity, elastase-1 levels, and pancreatic stiffness.

## 1. Introduction

Chronic pancreatitis (CP) is a disease of the pancreas in which recurrent inflammatory episodes result in replacement of pancreatic parenchyma by fibrous connective tissue which leads to progressive exocrine and endocrine pancreatic insufficiency [1–2]. Pancreatic exocrine insufficiency (PEI) results from insufficient secretion of pancreatic digestive enzymes and bicarbonate [2]. Steatorrhea, a clinically evident manifestation of PEI, occurs when the lipase output falls below 10 per cent of normal [3]. Similarly protein malabsorption may occur in chronic pancreatitis. Creatorrhea is diagnosed when daily fecal protein loss is > 2.5 g nitrogen and may occur in the late stages of chronic pancreatitis, when trypsin output is less than 10 per cent of normal [3]. However, protein digestion is continued by intestinal brush-border peptidases and is maintained even in the absence of luminal pancreatic proteolytic activity, thus creatorrhea is a late feature of CP [4]. In addition to PEI, other factors such as inflammation, anorexia, alcohol abuse and small intestinal bacterial overgrowth may contribute to protein malabsorption and altered amino acid levels in CP. Detectable alterations of essential nutrients, including amino acids may occur before symptoms or even before development of exocrine insufficiency [5]. Alterations in serum/plasma amino acid levels have been reported [5–8], but metabolomic studies of chronic pancreatitis are scarce [9–12]. The metabolome contains thousands of intermediates of varying physicochemical and biological properties making it difficult to include them all into a single experiment or discussion [13]. Therefore, here we report our findings related to the metabolism of proteinogenic amino acids in a metabolomics study of CP.

## 2. Materials and Methods

### 2.1. Study design and Population

This study was conducted in accordance with the Declaration of Helsinki of the World Medical Association and Institutional Review Board (IRB)/ Ethics Committee approval was obtained (Research Ethics Committee of the University of Tartu 278/T-22). All subjects provided written informed consent prior to inclusion in the study. No minors were included.

Twenty-five patients with chronic pancreatitis (CP) attending Tartu University Hospital from 29 January 2019–1 February 2022 were prospectively enrolled in this study. Given the exploratory nature of this study, the sample size reflects the availability of eligible participants during the enrolment period and was not based on a formal power calculation. Diagnosis of chronic pancreatitis was based on the M-ANNHEIM diagnostic criteria for chronic pancreatitis [14]. Patients with liver disease, inflammatory bowel disease, pancreatic or other organ malignancy were excluded from this study as well as patients with acute pancreatitis, an acute bout of chronic pancreatitis or a history of pancreatic surgery. Epidemiological data, accompanying disease and usage of medications, and alcohol intake and smoking were recorded. Height and weight were measured and body mass index (BMI) was calculated for all participants. Blood samples were collected after overnight fasting using Vacuette® CAT serum separator tubes. The samples were centrifuged at 4000 rpm and serum was frozen at −80°C until analysis.

Twenty eight age- and gender-matched healthy subjects satisfying the inclusion and exclusion criteria used in a previous study were included in a control group [15].

Pancreatic elastography was performed on consenting subjects during an endosonographic examination using the automatic elastography program with a PENTAX MEDICAL ultrasound endoscope (EG-3870UTK) and a Hitachi HI vision Avius ultrasound processor. For strain-ratio calculation, two regions of interest were selected: pancreatic parenchyma and a soft reference area outside the pancreatic tissue. The strain ratio was calculated by dividing the elastography result of the pancreatic parenchyma by the strain value of the reference soft tissue. Three measurements were performed in each anatomical region (head, body, and tail) whenever feasible. Fewer regions were assessed when technical limitations prevented reliable measurements (e.g., severe pancreatic tail atrophy, duodenal compression in chronic pancreatitis causing excessive transducer pressure and potentially artifactually elevated strain values).

For assessment exocrine pancreatic insufficiency, the determination of elastase-1 in stool (monoclonal enzyme-linked immunoassay) was used.

### 2.2. Measurement of amino acids

The amino acids were quantified with MxP Quant 500 kit (Biocrates Life Sciences AG, Austria) on a Xevo TQ-XS linked to Acquity UPLC (Waters, USA) according to the kit manufacturer’s protocol. In brief, the samples were transferred onto filter papers preloaded with internal standards. After drying, all compounds containing an amino group were derivatised with phenylisothiocyanate and dried again. Finally, the metabolites were extracted with 5 mM ammonium acetate in methanol and subjected to liquid chromatography-mass spectrometry analysis.

### 2.3. Statistical analysis

Statistical analyses were carried out using R-4.2.2. No missing data occurred for amino acid concentrations or demographic data. Not all patients did undergo elastography and the patients with missing values were excluded from the respective correlation analysis. Normal distribution of concentrations was not achieved for all amino acids even after log-transformation, therefore non-parametric Wilcoxon test was used to compare raw data and data adjusted to smoking. For demographic and clinical data chi-square, Wilcoxon or t-test was used depending on the data type.

A trend was defined as having an uncorrected p-value less than 0.05, but more than 0.0025. The latter is the estimated p-value below which the false positives due to multiple comparisons are controlled in our dataset. This value was calculated as suggested by Peluso A et al. [16]. Many non-significant trends appeared in biologically expected relationships; thus they were considered to be of interest.

## 3. Results

The patients with chronic pancreatitis (CP) and the control group had no significant differences regarding most clinical characteristics (Table 1). All but one CP patient were smokers, which was significantly more than in the control group.

**Table 1:** Characteristics of patients with chronic pancreatitis and controls^1^.

	Chronic pancreatitis (n = 25)	Controls (n = 28)	p-value	Test
Males (%)	5 (20%)	10 (35.7%)	0.33	Chi square
Age (quantiles)	55 (42-61)	60 (46-65)	0.33	Wilcoxon
Height (quantiles)	1.75 (1.68-1.81)	1.74 (1.71-1.79)	0.13	Student’s
Weight (quantiles)	70 (61-82)	76 (63-83)	0.56	Student’s
BMI^2^ (quantiles)	22.9 (21-26.6)	23.6 (21-27.4)	0.21	Wilcoxon
Hypertension (%)	14 (56%)	19 (68%)	0.54	Chi square
Diabetes (%)	11 (44%)	10 (35.7%)	0.74	Chi square
Smokers (%)	24 (96%)	5 (17.8%)	**5.6x10** ^**-8**^	Chi square
CRP^3^ (quantiles)	2 (0.7-5)	2 (0.7-7.6)	0.52	Wilcoxon

^1^Differences more significant than the false discovery rate corrected p-value are bold and underlined.

^2^Body Mass Index

^3^C-reactive Protein

The levels of proteinogenic amino acids were quantified and sums of amino acids by their structural or functional relationships were calculated (Table 2). A statistically significant decrease was detected for tryptophan and a significant increase was seen in the ratio of nonessential amino acids to essential amino acids, aspartate and glutamate levels. The term nonessential is used as common term for amino acids that the human body can synthesize itself. Additionally, several other amino acids had a tendency to be either decreased or increased in CP.

**Table 2:** Amino acids and calculated indexes (in italic) thereof.

	Chronic pancreatitis	Control	Unadjusted p-value	Smoking adjusted model p-value
**Amino acid sums, concentration µM**				
*Sum of all amino acids*	3450 (3049-4183)	3474 (3277-3834)	0.97	0.52
*Essential amino acids*	1051 (931-1295)	1198 (1068-1283)	0.083	**0.0132** ^¶^
*Nonessential amino acids*	2441 (2167-2781)	2337 (2121-2560)	0.41	0.84
*Solely glucogenic amino acids*	2851 (2481-3263)	2788 (2623-3075)	0.72	0.78
*Solely ketogenic amino acids*	320 (298-457)	418 (373-445)	0.13	**0.0079^¶^**
*Sulfur containing amino acids*	69 (56-97)	79 (72-96)	**0.036^¶^**	**0.011^¶^**
*Aromatic amino acids*	206 (162-258)	220 (199-248)	0.39	0.15
*Branched chain amino acids*	468 (412-542)	533 (470-565)	0.063	**0.020^¶^**
*Ratio of nonessential amino acid to essential amino acids*	2.35 (1.97-2.52)	2.0 (1.84-2.16)	**0.0020** ^†^	**0.0042^¶^**
**Proteinogenic amino acids, concentration µM**				
Alanine	522 (426-640)	501 (396-601)	0.44	1.0
Arginine	145 (120-191)	153 (126-181)	0.90	0.70
Asparagine	50 (40-62)	54 (49-64)	0.30	0.13
Aspartic acid	34 (24-66)	22 (18-28)	**0.00047^†^**	**0.016^¶^**
Cysteine	46 (41-56)	51 (46-69)	0.066	0.084
Glutamine	618 (477-746)	755 (658-838)	**0.025^¶^**	**0.017^¶^**
Glutamic acid	183 (140-335)	93 (71-110)	**0.000006^†^**	**0.00005^†^**
Glycine	268 (221-334)	265 (218-329)	0.88	0.62
Histidine	99 (89-106)	99 (86-108)	0.35	0.84
Isoleucine	89 (76-107)	95 (84-106)	0.22	0.26
Leucine	152 (117-184)	169 (147-179)	0.13	**0.032^¶^**
Lysine	188 (157-271)	244 (219-272)	**0.056^¶^**	**0.0062^¶^**
Methionine	22 (18-28)	28 (24-32)	**0.013^¶^**	**0.009^¶^**
Phenylalanine	74 (55-100)	72 (63-78)	0.80	0.99
Proline	297 (208-334)	240 (200-287)	0.086	0.23
Serine	152 (117-186)	135 (126-166)	0.79	0.54
Threonine	128 (105-142)	137 (126-165)	0.14	0.14
Tryptophan	61 (52-68)	70 (62-82)	**0.0056^¶^**	**0.0024^†^**
Tyrosine	70 (59-82)	72 (66-85)	0.25	0.28
Valine	240 (198-256)	268 (245-283)	**0.024^¶^**	**0.013^¶^**

Data median and 25–75% interquartile range are given.

¶ Differences with trends.

† Differences more significant than the false discovery rate corrected p-value.

**Fig 1:**
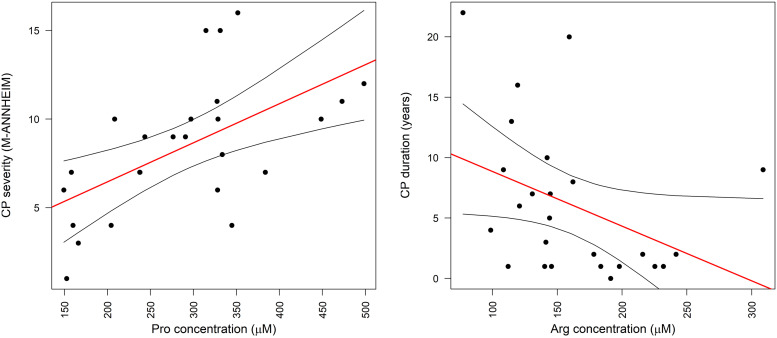
Correlation of chronic pancreatitis severity and disease duration with plasma amino acid concentrations. Serum proline level (x-axis) correlation with the severity of CP (y-axis). Spearman r 0.63. Serum arginine level (x-axis) correlation with the duration of CP (y-axis). Red line – linear regression of the relationship, black lines – 95% confidence intervals for the linear regression line.

Within the patient group, correlations between the level of amino acids and CP duration, severity, elastase-1 levels and elastography results were calculated. Pancreas head elastography data were available for 13, and body elastography for 14 patients out of 25. Weak to average positive correlation (0.18–0.60) was found for all proteinogenic amino acids with pancreatitis severity (M-ANNHEIM). The strongest correlation was with proline (Fig 1). The correlation with severity was generally stronger with nonessential amino acids; also the summary concentration of nonessential amino acids (r = 0.63, p < 0.001) displayed a stronger relationship than the sum of essential amino acids (0.35, p = 0.08). The sum of glucogenic amino acids (r = 0.63, p < 0.001) had a stronger association with severity than the sum of ketogenic amino acids (r = 0.38, p = 0.06).

**Fig 2:**
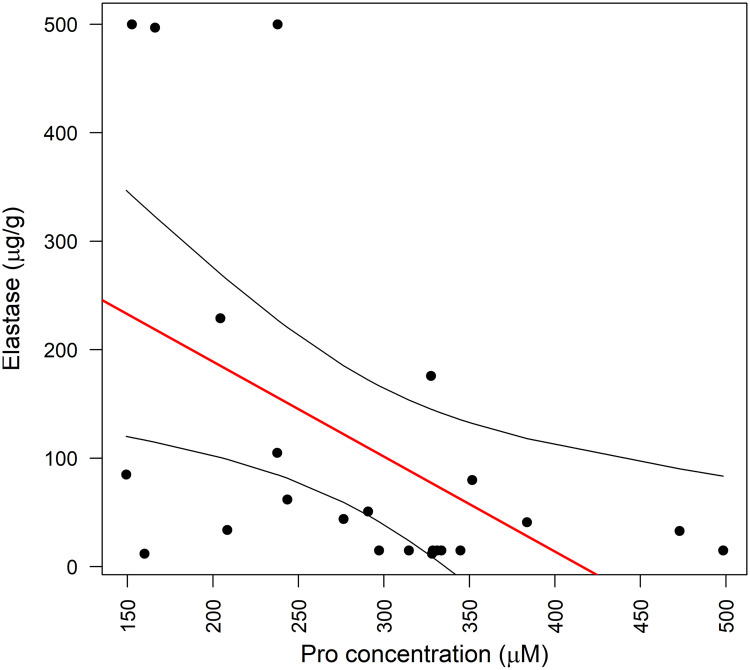
Correlation between fecal elastase-1 levels and plasma proline concentration. Serum proline concentration (x-axis) and fecal elastase (y-axis). r = −0.48, p = 0.02.

The duration of CP had less consistent relationships with amino acid levels. Arginine and cysteine had negative correlation with duration (r = −0.43, p = 0.03) (Fig 1). Other amino acids had no statistically significant or insignificant but at least moderate correlation. Elastase-1 correlated significantly only with proline (r = −0.48, p = 0.02) (Fig 2).

**Fig 3:**
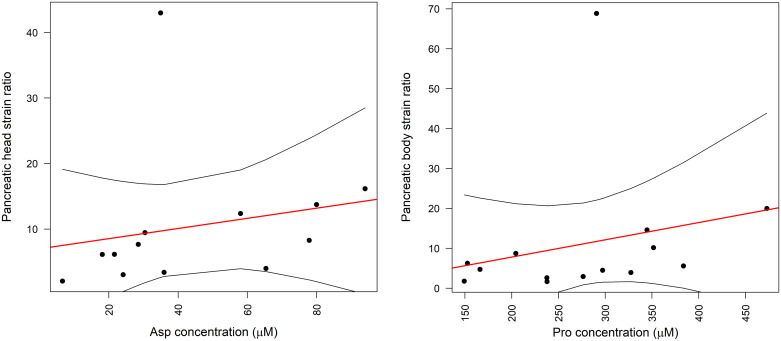
Associations between pancreatic strain ratio and serum amino acid concentrations in chronic pancreatitis. Left: dependence of pancreas head strain ratio on serum aspartate concentration (r = 0.60, p = 0.03) Right: dependence of pancreas body strain ratio on serum proline concentration (r = 0.47, p = 0.09).

Statistically significant correlation was found between aspartic acid and pancreas head elastography (r = 0.60, p = 0.03). (Fig 3). There was no correlation between aspartic acid and pancreas body elasticity. Although statistically insignificant, an average strength correlation with pancreas head elasticity was found for arginine, glutamic acid and glycine (all r = 0.41, p = 0.16). Pancreas body elasticity measurements had average correlation with serum proline (r = 0.47, p = 0.09, Fig 3) and tyrosine (r = 0.40, p = 0.16) levels.

## 4. Discussion

Chronic pancreatitis is still lacking good diagnostic biomarkers. Both the exocrine and endocrine functions of the pancreas regulate management of nutrients – from digestion and absorption to usage or storage. Therefore, the profile of metabolites in the serum could be significantly shifted in chronic pancreatitis.

Amino acids can be classified in different ways based on their structural features and by their role in metabolism. Proteinogenic amino acids are central to protein metabolism. They also serve as substrates for more specific pathways that lead to synthesis of signaling molecules (e.g., histamine, serotonin) or other bioactive compounds (e.g., carnitine, taurine, niacin, glutathione). This makes them suitable markers for studies investigating overall amino acid metabolism or screening for potentially important aberrations in amino acid related pathways without having a specific preliminary hypothesis.

In the current study the total concentration of proteinogenic amino acids in CP patients was not different from the control group. Non-significant trends were observed in subgroups. The sum of essential amino acids, which cannot be synthesized by body, had a tendency to be lower in CP. In contrast the ratio of non-essential to essential amino acids was significantly higher in CP patients. These findings align with earlier reports indicating a tendency for decreased concentrations of total and essential amino acids in patients with CP [5–8]. Statistical significances do not appear in all studies, but it should be noted that most of the CP metabolomic studies, including ours, thus far have relatively small study groups.

Essential amino acids originate from diet but breakdown of body proteins also makes a certain contribution. Although both are possible, in the case of CP, reduced availability of dietary amino acids is more plausible. Gastric, pancreatic and ileal brush border enzymes are needed to break dietary proteins into individual amino acids. Exocrine insufficiency of pancreas removes the main enzymes responsible for cutting denaturated protein chains into shorter oligomers, which are further degraded by brush border enzymes. By skipping pancreatic enzymes only a fraction of dietary amino acids is absorbed [17–18].

Increasing severity of CP was found to increase the level of nonessential amino acids. This may indicate increased breakdown of body protein to cover the need for essential amino acids for pathways other than protein synthesis. A significant association with severity was also found when amino acids were divided based on their gluco- or ketogenic nature. There is a large overlap between glucogenic and essentiality-based classifications obscuring the potential reasons why amino acids could respond as a group. Loss of pancreatic insulin could drive the need for glucogenic substances, because gluconeogenesis is not suppressed anymore. However, accumulation of glucogenic amino acids despite increased use indicates a greater inflow compared to their consumption. This inflow can be either increased breakdown of body protein reserves or increased absorption from the diet. Thus, a shift in protein breakdown seems to be a major metabolic consequence of more severe CP, while the use of individual amino acids is more conditional. CP duration did not correlate with most individual or group-wise amino acid levels, which implies that deterioration of protein reserves is avoidable if CP is stable and controlled.

The differences between individual amino acids in CP should not be overlooked. Tryptophan, lysine, methionine, and valine were individually lower in CP patients, whereas aspartic acid and glutamic acid were increased. Aspartic acid, alanine and glutamic acid are involved in transamination, which is a key process in linking metabolism of all amino acids to carbohydrate metabolism and cellular energetics. Their concentration may rise if protein degradation or amino acid catabolism increases. Increased blood concentrations of glutamic acid in chronic pancreatitis have also been reported with alcoholic and tropical pancreatitis [5–8].

An interesting study with elemental diet found that glutamic acid concentration in CP decreased after the diet while the concentration of essential amino acids (methionine and histidine in this study) increased [19]. This further supports the hypothesis that increase in glutamic acid is related to consumption of other amino acids.

Alterations in proline levels in CP have been reported before with both increases [6] and decreases described [7]. Differences in age, comorbidities and other baseline characteristics can be noted when comparing the studies with controversial reports on proline. Metabolically, proline is directly associated with glutamic acid, increase in which has a better consensus. Girish *et al* suggest that severe exocrine deficiency reduces the body’s ability to increase serum proline levels [6]. It could be that competing processes can increase or decrease proline levels in CP, and it comes to fine details as to which effect prevails. Moreover, in our study and in the study by Adrych *et al* the change in proline concentration was not robust but on the borderline [7]. We consider proline nonetheless to be of special interest due to the correlations with CP severity and elastography results.

Although proline was not significantly elevated in CP patients, it showed the strongest associations with CP severity, fecal elastase-1 levels and elastography results. Proline and its derivative, hydroxylated proline, make up approximately 23% of collagen, which is a major connective tissue protein responsible for wound healing and some aspects of cirrhosis [20]. As recently reviewed, the proline needed for collagen synthesis can originate from protein breakdown or through *de novo* synthesis from other amino acids – glutamine in particular [21]. High proline levels in the cellular environment may stimulate fibroblasts to produce more collagen [22]. The fecal elastase level depends on the quantity of undamaged and thus functional exocrine cells. Increased damage results in functional tissue being replaced by connective tissue (e.g., mostly collagen). EUS-elastography offers a quantitative assessment of tissue stiffness. In chronic pancreatitis, the stiffness of the pancreatic parenchyma is primarily attributed to fibrosis. A correlation has been demonstrated between the degree of pancreatic fibrosis, measured by EUS-elastography, and the likelihood of PEI in patients with chronic pancreatitis. Pancreatic function declines linearly as pancreatic fibrosis increases [23–24]. Associations of serum proline with fecal elastase and pancreas strain ratios are therefore anticipated results.

Fibrosis and collagen synthesis may also explain some other changes observed. Collagen is not as diverse in its amino acid composition as most proteins. The most abundant essential amino acids in collagen molecules are lysine, threonine, valine and leucine [20]. Their essentiality and requirement for collagen synthesis might render them the amino acids most susceptible to deficiency in CP. The decreases in tryptophan and methionine in CP are less likely related to collagen synthesis and would require additional studies for explanation.

In addition to disease-related mechanisms, important confounding factors must be considered when interpreting our findings. The prevalence of smoking differed markedly between the groups. Cigarette smoking can influence circulating amino-acid profiles and related metabolic pathways. Smoking induces oxidative stress and systemic inflammation, leading to altered levels of essential amino acids such as tryptophan [25–26] and methionine [27], increased turnover of glutamate, glycine, and cysteine through glutathione-related pathways [28], and disruption of arginine–nitric oxide metabolism [29]. Adjustment for smoking did not cause major changes in our outcomes, but the small sample size sets limits to the reliability of statistical adjustments.

Dietary variability, which could be another important confounder, was not assessed, and should be included in future studies.

## Limitations

Our study has several limitations. First, the study had a relatively small sample size, and no formal power calculation was performed. Therefore, the analyses should be considered exploratory, and the statistical power to detect subtle metabolomic differences may be limited. The cross-sectional design prevents the establishment of causal relationships.

The pronounced imbalance in smoking status between groups represents a major limitation of this study, as smoking can independently influence amino-acid metabolism. Dietary variability was not assessed, which could confound amino-acid results.

## Conclusion

Taken together, our results show that CP is associated with significant deficiency in essential amino acids. Increased breakdown of proteins seems to occur in order to provide the essential amino acids. Increasing severity of CP appears to enhance the breakdown of other proteins to provide amino acids required for collagen synthesis, with proline showing the strongest associations with fecal elastase and the pancreatic strain ratio. Our study highlights the potential of proline as a biomarker of severity in CP. Further studies are needed in larger cohorts that include etiologically diverse forms of chronic pancreatitis, and longitudinal designs are important to validate proline as a potential biomarker in chronic pancreatitis.

## References

[1] Hoffmeister A, Mayerle J, Beglinger C, Büchler MW, Bufler P, Dathe K, et al. English language version of the S3-consensus guidelines on chronic pancreatitis: Definition, aetiology, diagnostic examinations, medical, endoscopic and surgical management of chronic pancreatitis. Z Gastroenterol. 2015;53(12):1447–95. doi: 10.1055/s-0041-107379 26666283

[2] Löhr JM, Dominguez-Munoz E, Rosendahl J, Besselink M, Mayerle J, Lerch MM, et al. United European Gastroenterology evidence-based guidelines for the diagnosis and therapy of chronic pancreatitis (HaPanEU). United European Gastroenterol J. 2017;5(2):153–99. doi: 10.1177/2050640616684695 28344786 PMC5349368

[3] DiMagno EP, Go VL, Summerskill WH. Relations between pancreatic enzyme outputs and malabsorption in severe pancreatic insufficiency. N Engl J Med. 1973;288(16):813–5. doi: 10.1056/NEJM197304192881603 4693931

[4] Meier RF, Beglinger C. Nutrition in pancreatic diseases. Best Pract Res Clin Gastroenterol. 2006;20(3):507–29. doi: 10.1016/j.bpg.2006.01.004 16782526

[5] Kawaguchi Y, Ogawa M, Ito H, Mine T. Alterations in plasma amino acid levels in alcoholic chronic pancreatitis in Japanese. Digestion. 2012;86(2):155–60. doi: 10.1159/000339781 22889918

[6] Girish BN, Rajesh G, Vaidyanathan K, Balakrishnan V. Alterations in plasma amino acid levels in chronic pancreatitis. JOP. 2011;12(1):11–8. 21206095

[7] Adrych K, Smoczynski M, Stojek M, Sledzinski T, Slominska E, Goyke E, et al. Decreased serum essential and aromatic amino acids in patients with chronic pancreatitis. World J Gastroenterol. 2010;16(35):4422–7. doi: 10.3748/wjg.v16.i35.4422 20845509 PMC2941065

[8] Schrader H, Menge BA, Belyaev O, Uhl W, Schmidt WE, Meier JJ. Amino acid malnutrition in patients with chronic pancreatitis and pancreatic carcinoma. Pancreas. 2009;38(4):416–21. doi: 10.1097/MPA.0b013e318194fc7a 19169171

[9] Díaz C, Jiménez-Luna C, Diéguez-Castillo C, Martín A, Prados J, Martín-Ruíz JL, et al. Untargeted Metabolomics for the Diagnosis of Exocrine Pancreatic Insufficiency in Chronic Pancreatitis. Medicina. 2021;57(9):876. doi: 10.3390/medicina57090876 34577799 PMC8470962

[10] Adam MG, Beyer G, Christiansen N, Kamlage B, Pilarsky C, Distler M, et al. Identification and validation of a multivariable prediction model based on blood plasma and serum metabolomics for the distinction of chronic pancreatitis subjects from non-pancreas disease control subjects. Gut. 2021;70(11):2150–8. doi: 10.1136/gutjnl-2020-320723 33541865 PMC8515121

[11] Lindahl A, Heuchel R, Forshed J, Lehtiö J, Löhr M, Nordström A. Discrimination of pancreatic cancer and pancreatitis by LC-MS metabolomics. Metabolomics. 2017;13(5):61. doi: 10.1007/s11306-017-1199-6 28413374 PMC5376388

[12] Wu L, Huang X, Ouyang Q, Liu W, Liu S, Huang Y, et al. Serum metabolomics study for acute attack of chronic pancreatitis. Clinica Chimica Acta. 2023;541:117251. doi: 10.1016/j.cca.2023.117251 36775008

[13] Wishart DS. Metabolomics for Investigating Physiological and Pathophysiological Processes. Physiol Rev. 2019;99(4):1819–75. doi: 10.1152/physrev.00035.2018 31434538

[14] Schneider A, Löhr JM, Singer MV. The M-ANNHEIM classification of chronic pancreatitis: introduction of a unifying classification system based on a review of previous classifications of the disease. J Gastroenterol. 2007;42(2):101–19. doi: 10.1007/s00535-006-1945-4 17351799

[15] Müller R, Kull M, Lember M, Põlluste K, Valner A, Kallikorm R. Insulin Resistance in Early Rheumatoid Arthritis Is Associated with Low Appendicular Lean Mass. Biomed Res Int. 2017;2017:9584720. doi: 10.1155/2017/9584720 28932748 PMC5592389

[16] Peluso A, Glen R, Ebbels TMD. Multiple-testing correction in metabolome-wide association studies. BMC Bioinformatics. 2021;22(1):67. doi: 10.1186/s12859-021-03975-2 33579202 PMC7881646

[17] Mary F, Moesseler A, Khodorova N, Foucault-Simonin A, Benamouzig R, Tomé D, et al. Metabolic markers of protein maldigestion after a 15N test meal in minipigs with pancreatic exocrine insufficiency. Am J Physiol Gastrointest Liver Physiol. 2018;314(2):G223–30. doi: 10.1152/ajpgi.00218.2017 29074486

[18] Airinei G, Gaudichon C, Bos C, Bon C, Kapel N, Bejou B, et al. Postprandial protein metabolism but not a fecal test reveals protein malabsorption in patients with pancreatic exocrine insufficiency. Clin Nutr. 2011;30(6):831–7. doi: 10.1016/j.clnu.2011.06.006 21741734

[19] Kawaguchi Y, Lin J-C, Kawashima Y, Maruno A, Ito H, Ogawa M, et al. Relationship between pain and plasma amino acid levels in chronic pancreatitis. JOP. 2015;16(1):53–7. doi: 10.6092/1590-8577/2897 25640784

[20] Albaugh VL, Mukherjee K, Barbul A. Proline Precursors and Collagen Synthesis: Biochemical Challenges of Nutrient Supplementation and Wound Healing. J Nutr. 2017;147(11):2011–7. doi: 10.3945/jn.117.256404 28978679 PMC5657141

[21] Karna E, Szoka L, Huynh TYL, Palka JA. Proline-dependent regulation of collagen metabolism. Cell Mol Life Sci. 2020;77(10):1911–8. doi: 10.1007/s00018-019-03363-3 31740988 PMC7228914

[22] Szoka L, Karna E, Hlebowicz-Sarat K, Karaszewski J, Palka JA. Exogenous proline stimulates type I collagen and HIF-1α expression and the process is attenuated by glutamine in human skin fibroblasts. Mol Cell Biochem. 2017;435(1–2):197–206. doi: 10.1007/s11010-017-3069-y 28526934 PMC5632346

[23] Iglesias-Garcia J, Domínguez-Muñoz JE, Castiñeira-Alvariño M, Luaces-Regueira M, Lariño-Noia J. Quantitative elastography associated with endoscopic ultrasound for the diagnosis of chronic pancreatitis. Endoscopy. 2013;45(10):781–8. doi: 10.1055/s-0033-1344614 24019131

[24] Dominguez-Muñoz JE, Iglesias-Garcia J, Castiñeira Alvariño M, Luaces Regueira M, Lariño-Noia J. EUS elastography to predict pancreatic exocrine insufficiency in patients with chronic pancreatitis. Gastrointest Endosc. 2015;81(1):136–42. doi: 10.1016/j.gie.2014.06.040 25088920

[25] Pertovaara M, Heliövaara M, Raitala A, Oja SS, Knekt P, Hurme M. The activity of the immunoregulatory enzyme indoleamine 2,3-dioxygenase is decreased in smokers. Clin Exp Immunol. 2006;145(3):469–73. doi: 10.1111/j.1365-2249.2006.03166.x 16907915 PMC1809700

[26] Onmaz M, Eryavuz Onmaz D, Demirbas N, Kutlu R, Unlu A, Hatir AE. Tobacco induces abnormal metabolism of tryptophan via the kynurenine pathway. Turkish Journal of Biochemistry. 2025;50(1):152–9. doi: 10.1515/tjb-2024-0286

[27] Aslam MA, Iqbal H, Ilyas K, Rehman K, Hussain A, Akash MSH, et al. Metabolomic Insights into Smoking-Induced Metabolic Dysfunctions: A Comprehensive Analysis of Lipid and Amino Acid Metabolomes. Metabolites. 2025;15(2):96. doi: 10.3390/metabo15020096 39997721 PMC11857658

[28] Moriarty SE, Shah JH, Lynn M, Jiang S, Openo K, Jones DP, et al. Oxidation of glutathione and cysteine in human plasma associated with smoking. Free Radic Biol Med. 2003;35(12):1582–8. doi: 10.1016/j.freeradbiomed.2003.09.006 14680681

[29] Zhang W-Z, Venardos K, Chin-Dusting J, Kaye DM. Adverse effects of cigarette smoke on NO bioavailability: role of arginine metabolism and oxidative stress. Hypertension. 2006;48(2):278–85. doi: 10.1161/01.HYP.0000231509.27406.42 16801489

